# Fabrication of Nanoscale Gas–Liquid Interfaces in Hydrophilic/Hydrophobic Nanopatterned Nanofluidic Channels

**DOI:** 10.1021/acs.nanolett.1c02871

**Published:** 2021-10-14

**Authors:** Hiroto Kawagishi, Shuichi Kawamata, Yan Xu

**Affiliations:** † Department of Chemical Engineering, Graduate School of Engineering, Osaka Prefecture University, 1-2, Gakuen-cho, Naka-ku, Sakai, Osaka 599-8570, Japan; ‡ Department of Quantum and Radiation Engineering, Graduate School of Engineering, Osaka Prefecture University, 1-2, Gakuen-cho, Naka-ku, Sakai, Osaka 599-8570, Japan; § Japan Science and Technology Agency (JST), PRESTO, 4-1-8 Honcho, Kawaguchi, Saitama 332-0012, Japan; ∥ NanoSquare Research Institute, Research Center for the 21st Century, Organization for Research Promotion, Osaka Prefecture University, 1-2, Gakuen-cho, Naka-ku, Sakai, Osaka 599-8570, Japan

**Keywords:** nanoscale gas−liquid interfaces, nanofluidic devices, nanopatterned nanochannels, nanoarray, nano-in-nano integration, manipulation, enrichment

## Abstract

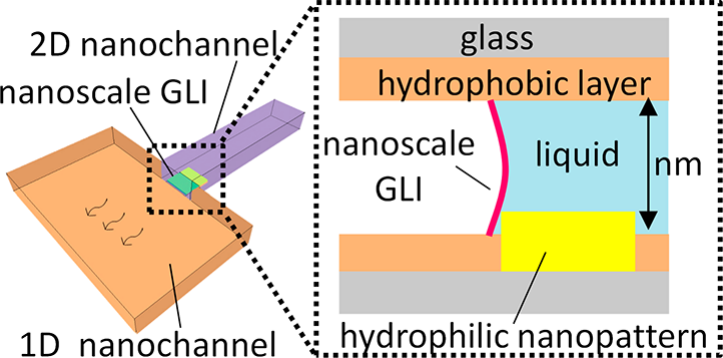

Gas–liquid interfaces (GLIs) are ubiquitous and have found widespread applications in a large variety of fields. Despite the recent trend of downscaling GLIs, their nanoscale fabrication remains challenging because of the lack of suitable tools. In this study, a nanofluidic device, which has undergone precise local surface modification, is used in combination with tailored physicochemical effects in nanospace and optimized nanofluidic operations, to produce uniform, arrayable, stable, and transportable nanoscale GLIs that can concentrate molecules of interest at the nanoscale. This approach provides a delicate nanofluidic mechanism for downscaling gas–liquid interfaces to the nanometer scale, thus opening up a new avenue for gas–liquid interface studies and applications.

Regardless of nature or engineering, gas–liquid interfaces (GLIs) play an important role in numerous chemical and biological processes (e.g., solute enrichment, chemical reactions, dispensation, separation, and mass/energy transfer).^1−3^ In recent years, there is a trend to downscale GLIs to the microscale and nanoscale for expanding their potentials, and microscale GLIs have been studied due to the availability of numerous fabrication methods for using surface patterned substrates,^4,5^ inkjets,^6^ and microfluidic devices.^1^ Nanoscale GLIs have been randomly generated in carbon nanotubes,^7^ porous membranes,^8^ sonicated solutions,^9^ and sprays.^10^ However, the fabrication of controllable nanoscale GLIs for being applied in the aforementioned processes is still challenging because of the lack of tools suitable for handling nanoscale fluids.

Nanofluidics is the study and application of fluids in nanoscale environments. Chip-based nanofluidic devices (hereafter referred to as “nanofluidic devices”) commonly used in this field can manipulate fluids in nanometer-scale channels with a precisely controlled geometry,^11−16^ and they have the potential to fabricate controllable nanoscale GLIs. However, to fabricate such GLIs using nanofluidic devices, the properties of the channel surface, especially the wettability, need to be controlled. Unfortunately, nanofluidic channels are quite small to make use of the conventional approach of surface control. For example, surface patterning combined with photolithography is commonly used to fabricate microscale GLIs in microfluidic channels, but it does not have a spatial resolution sufficiency for the fabrication of functional surfaces in nanofluidic channels due to diffraction, photomask resolution, and the accuracy of instrumental operations. Diffusion-limited patterning can control the surface of a nanofluidic channel by controlling the reactive solute molecules diffusing in the solution.^17^ However, the practical spatial resolution of this method is limited by the size of the channel, reaction rate, diffusion coefficient, and nanofluidic operation. Hence, to fabricate controllable nanoscale GLIs, special surface control strategies are required to control the wettability of nanofluidic channels with a nanometer-scale spatial resolution.

In this study, we demonstrate the fabrication of controllable nanoscale GLIs in femtoliter-order nanofluidic channels by a combination of precise local surface control, tailored physicochemical effects in nanospace, and optimized nanofluidic operations. Our approach involves the formation of a nanoscale GLI induced by the local phase change occurring in hydrophobic/hydrophilic nanopatterned nanofluidic channels. As a result, the fabrication of nanoscale uniform, stable, arrayable nanoscale GLIs in the tiny nanofluidic channels was achieved. Furthermore, the fabricated nanoscale GLIs can be manipulated through nanofluidic channels and possess the ability to concentrate molecules of interest (e.g., rhodamine B) at the nanoscale, suggesting the potential to be applied in chemical, physical, and biological processes.

Figure 1 depicts the concept underlying the fabrication of nanoscale GLIs applied in this study by using hydrophilic/hydrophobic nanopatterned nanofluidic channels. The depth of the channels *d* is uniform and is of nanoscale. The width of the channels *w* has a micrometer-scale-wide part (hereinafter called one-dimensional (1D) nanofluidic channels) in the left region and a narrow nanoscale-wide part (hereinafter called two-dimensional (2D) nanofluidic channels) from the middle to the right region of the channels. The surface of the channel is hydrophobic, except for the entrance area of the 2D nanofluidic channels that have hydrophilic nanopatterns. In this case, the change of vapor pressure in the nanofluidic channels can be described by the Kelvin equation.^18^
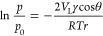
where *p*, *p*_0_, *V*_L_, γ, and θ denote the actual vapor pressure of the liquid in the nanofluidic channels, saturated vapor pressure of the bulk liquid, molar volume of the liquid, surface tension of the liquid, and contact angle between the liquid and surface of the nanofluidic channels, respectively; *R* is the gas constant; *T* is the temperature; and *r* is the hydraulic radius of the channels. The hydraulic radius of the rectangular channels can be defined as *wd*/(*w* + *d*).^19,20^ The equation suggests that *r* and θ affect *p*, because the interaction between the solid surface and liquid molecules is not negligible in a small space compared to a bulk space. While *p*/*p*_0_ for the 1D hydrophobic nanofluidic channels (θ > 90°, *r* is large) is quite larger than 1, *p*/*p*_0_ for the 2D hydrophilic nanofluidic channels (θ < 90°, *r* is small) is smaller than 1. Further considering the large difference in *r* between the 1D nanofluidic channels and the 2D nanofluidic channels, *p* in the 1D hydrophobic nanofluidic channels (*r* is large) would be significantly higher than that in the 2D hydrophilic nanofluidic channels (*r* is small). This suggests that the liquid in the 1D hydrophobic nanofluidic channels evaporates more easily than the liquid in the 2D hydrophilic nanofluidic channels. In other words, *p* in the 1D hydrophobic nanofluidic channels is high enough to easily cause the phase changes resulting from the combined effects of θ and *r*. Therefore, the nanoscale GLIs may form along the borders between the 1D and 2D nanofluidic channels as a result of the phase changes.

**Figure 1:**
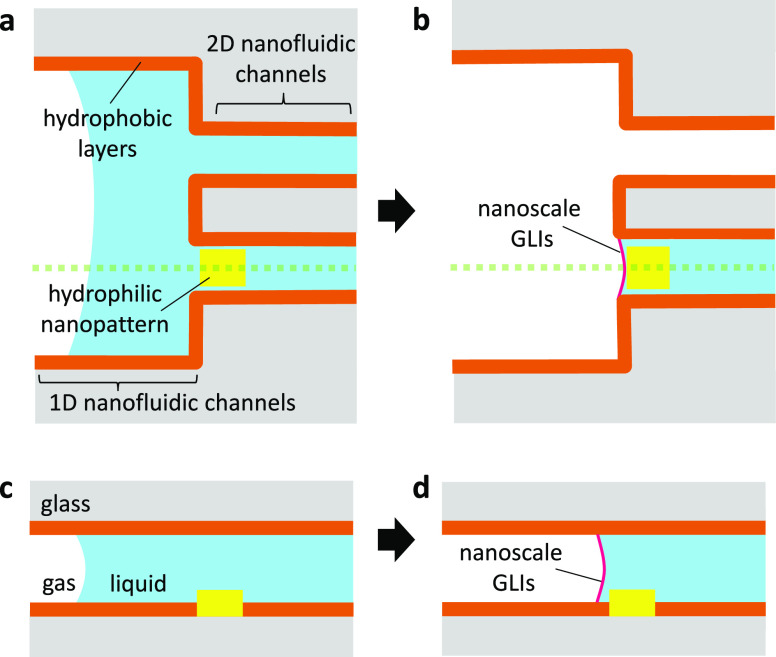
Conceptual schematic of fabrication of nanoscale GLIs in hydrophilic/hydrophobic nanopatterned nanofluidic channels. Cross sections at the plane of hydrophilic nanopatterns of the nanofluidic channels (a) before and (b) after the fabrication of nanoscale GLIs. Longitudinal sections along the green dotted line in (a) and (b) of the nanofluidic channels (c) before and (d) after the fabrication of nanoscale GLIs, respectively.

Based on the schematic shown in Figure 1, a nanofluidic device with hydrophilic/hydrophobic nanopatterns was fabricated and applied to construct controllable nanoscale GLIs (Figure 2a,d). The parallel-arrayed 2D nanofluidic channels (10 channels, 860 nm in width, 180 nm in depth, 100 μm in length) were located between the 1D nanofluidic channels (630 μm in width, 180 nm in depth, 500 μm in length) (Figures 2b and S1a). The gold nanopatterns (5 nanopatterns, 500 nm in width, 50 nm in height, 460 nm in length) were positioned at the end of the five 2D nanofluidic channels with an accuracy on the scale of tens of nanometers (Figures 2c,e and S1b). The gold nanopatterned nanofluidic channels were fabricated using a nano-in-nano integration technology developed by us previously.^20−22^ The 2D nanofluidic channels without gold nanopatterns were used for control experiments. After the device fabrication, the hydrophilic/hydrophobic nanopatterns were obtained by the selective silanization process performed in the nanofluidic channels. The nanofluidic channels and nanopatterns were made of glass and gold, respectively, and both the original surfaces were hydrophilic (Figure 2f). The hydrophilic/hydrophobic nanopatterns were obtained by silanizing trichloromethylsilane^23^ as hydrophobic molecules to the glass surface of the nanofluidic channels and rinsing them thereafter (Figure 2f). Such surface modification is commonly used for open substrates and microfluidic channels, but its applicability for nanofluidic channels is almost unexplored. Therefore, we compared the conditions of surface modification for nanofluidic channels. The nanofluidic channels were silanized and rinsed by using a liquid introduction system, which has been previously reported by us^24,25^ (the details of the methods are described in the Supporting Information, Figures S2 and S3). After the surface modification, water was introduced into the nanofluidic channels, by applying external pressure, to verify whether the channels were silanized. Generally, water can naturally flow into bare nanochannels by capillary action without applying external pressure owing to the hydrophilic property of the glass surface of the channel wall. In contrast, we observed that water flow stopped at the entrances of all the 2D nanofluidic channels up to the external pressure of 400 kPa, and water could further flow into the 2D nanofluidic channels above 400 kPa (Figure S4). This result suggests that the hydrophobizing 2D nanofluidic channels were achieved by silanization. An external pressure is required for the introduction of water into the hydrophobic channels, because the Laplace pressure works in the opposite direction of introduction. Laplace pressure is the difference in pressure between the inside and the outside of GLIs in channels arising due to the surface tension. Here, the evaluation of surface wettability of the channel wall and the gold nanopatterns in nanofluidic channels is necessary to confirm such locally selective hydrophilic/hydrophobic surface modification. However, a contact angle meter, which is widely used for the evaluation of wettability, cannot be applied to closed spaces, making direct measurements of wettability in nanofluidic channels difficult. Therefore, we measured the contact angles on glass substrates and gold-coated substrates instead of those on the surface of the nanofluidic channels. The substrates were silanized and rinsed by immersing in toluene, ethanol, and a 1:1 mixture of ethanol and ultrapure water (details of methods in the Supporting Information). The contact angles of the glass and gold surfaces were measured before surface modification but having undergone silanization and rinsing, after the surface modification, and after the surface modification with a rinse in toluene for 24 h (Figure 2g). Both the glass and gold surfaces changed from hydrophilic (θ < 90°) (Figure S5a,b) to hydrophobic (θ > 90°) (Figure S5c,d) after surface modification, which is attributed to the nonspecific physical adsorption of trichloromethylsilane on the gold surface. In contrast, the gold surface, after silanization with the special rinse, was hydrophilic, and the glass counterpart was hydrophobic (Figures S5e,f), suggesting that the nonspecific physical adsorption was removed from the gold surface by diffusion of trichloromethylsilane into toluene during the special rinse. This resulted in a large difference in the contact angle between the glass and the gold surfaces, suggesting the formation of hydrophilic/hydrophobic nanopatterns in the nanofluidic channels.

**Figure 2:**
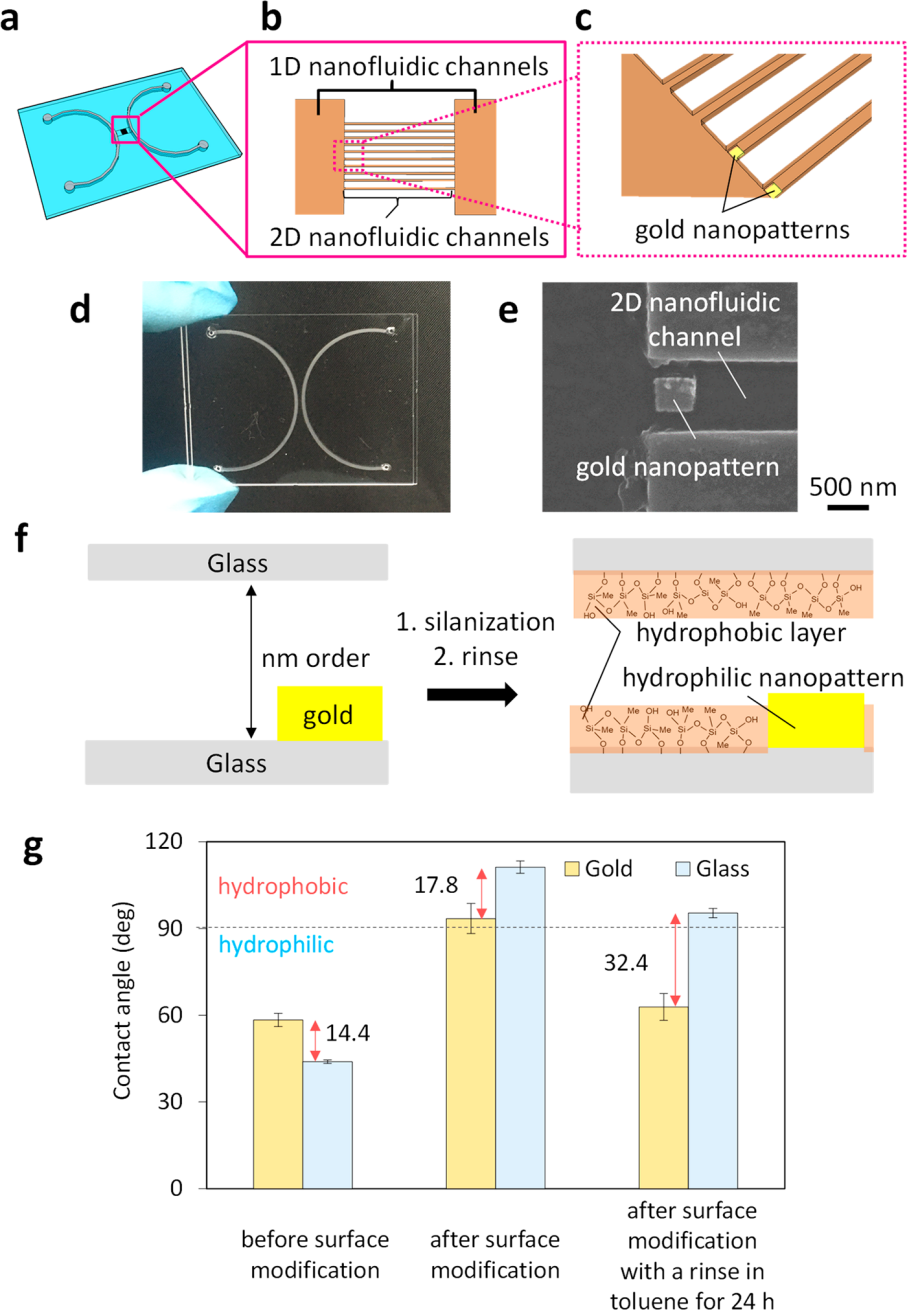
Schematic images of (a) the nanofluidic device, (b) 2D nanofluidic channels between 1D nanofluidic channels, and (c) hydrophilic/hydrophobic nanopatterns in the nanofluidic channels. (d) A digital image of the fabricated nanofluidic device. (e) A scanning electron microscopy (SEM) image of a nanofluidic channel with a gold nanopattern. (f) Schematic images of surface modification to obtain hydrophilic/hydrophobic nanopatterned nanofluidic channels. (g) Contact angles on the surface of gold and glass.

Nanoscale GLIs were fabricated in the nanofluidic device by a fine fluid control process described in Figure S6 in the SI. Figure 3a–c shows bright-field microscopic images of the nanofluidic channels throughout the nanoscale GLI fabrication process. First, 50% aqueous ethanol was introduced under an external pressure of 500 kPa to completely fill the nanofluidic channels by the liquid introduction system (Figure 3b). Air was then introduced under an external pressure of 200 kPa to remove the liquid from the left microchannel. After the liquid was removed, the liquid in the 1D nanofluidic channels gradually moved from the left side of the microfluidic channel in the direction of the 2D nanofluidic channel under zero external pressure. The nanoscale GLIs traveled to the 2D nanofluidic channels and uniformly stopped at the entrance of the 2D nanofluidic channels partially modified with gold nanopatterns. Although the liquid disappeared in the 2D nanofluidic channels without gold nanopatterns, the arrayed nanoscale GLIs were stable in the partially modified 2D nanofluidic channels for over 1 h (Figure 3c). This result implies that the difference in the actual vapor pressure between hydrophilic and hydrophobic areas in the 2D nanofluidic channels provided sufficient capability to keep the stability of the nanoscale GLIs for a long time. Such long-term stability will be very favorable to develop a variety of applications in the future. Luminous intensity of the hydrophilic/hydrophobic nanopatterns in 2D nanofluidic channels, as shown in Figure 3d–f, was plotted to confirm the uniformity in the fabricated nanoscale GLIs (Figure 3g). Differences in the luminous intensity between the blue and red lines were observed because of the difference in the refractive index of liquid and air. The black line (nanoscale GLI) has the same intensity as the red line (dry nanofluidic channels) in 1D nanofluidic channels and the same intensity as the blue line (liquid-filled nanofluidic channels) in 2D nanofluidic channels. This suggests that nanoscale GLIs were fabricated between the 1D and 2D nanofluidic channels. Gaps of 150 nm in size were allowed to be introduced between the gold nanopatterns and the edges of the 2D nanofluidic channels to align the nanopatterns in the 2D nanofluidic channels. Nevertheless, the nanoscale GLIs were also formed in these 2D nanofluidic channels, as the gaps were quite small to affect the difference in the vapor pressure. This was supported by the result that the liquid remained in the 2D nanofluidic channels with the gold nanopatterns, even though it disappeared in the 2D nanofluidic channels without the gold nanopatterns.

**Figure 3:**
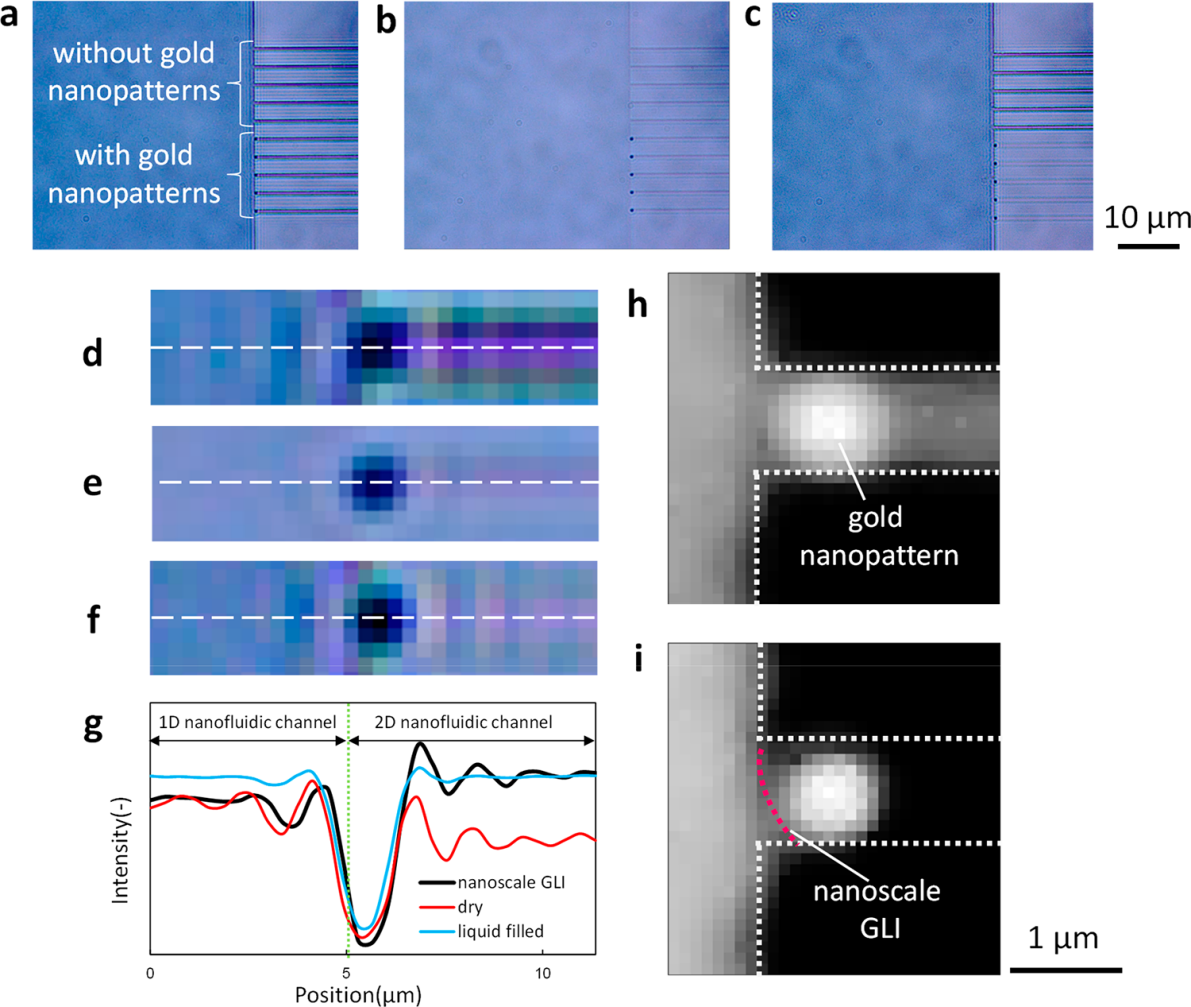
Fabrication of nanoscale GLIs in hydrophilic/hydrophobic nanopatterned nanofluidic channels. Microscopic images of (a) dry nanofluidic channels, (b) liquid-filled nanofluidic channels, (c) fabrication of nanoscale GLIs in the nanopatterned nanofluidic channels with the (d–f) scaling images of hydrophilic/hydrophobic nanopatterns in (a–c). (g) Luminous intensity profile along the white line in (d–f). Grayscale reflected light images of (h) the gold nanopattern in 2D nanofluidic channels and (i) the fabricated nanoscale GLI.

Furthermore, the positions of the nanoscale GLIs were characterized by reflected light microscopy. Figure 3h shows the reflected light images of the dry 2D nanofluidic channel with the gold nanopattern, while Figure 3i shows the fabricated GLIs in the 2D nanofluidic channel. As the difference in the luminous intensity between Figures 3h and 3i indicates the liquid phase, the location of nanoscale GLIs was confirmed by referring to the image of the difference in luminous intensity between two images generated using ImageJ (Figure S7). The red broken line shows the nanoscale GLI and is located within 300 nm from the gold nanopatterns, revealing that GLIs were positionally controlled by nanopatterns with a nanoscale accuracy. These results indicate that the fabricated nanoscale GLIs were uniform, stable, arrayable, and position-controlled by hydrophilic/hydrophobic nanopatterned nanofluidic channels.

The fabricated nanoscale GLIs could be manipulated by operating the external pressure ranging from 100 to 500 kPa. The response time of the manipulation was defined as the time between fabricating and moving the nanoscale GLIs. Figure 4a shows the response time measured in the 2D nanofluidic channels with different external pressures. This result indicates that the response time decreased as the external pressure increased and that the movement of nanoscale GLIs could start within a few seconds by applying an external pressure. The velocity of the movement was calculated from the moving distance of a nanoscale GLI in 2.6 s, which is equivalent to 20 movie frames (Figure 4b). Figure 4c shows that the velocity is linearly correlated with the external pressure. In the case of no external pressure, the GLIs remained on the gold nanopatterns for over 1 h (Figure 3c), suggesting that the movement of nanoscale GLIs can be controlled by an external pressure.

**Figure 4:**
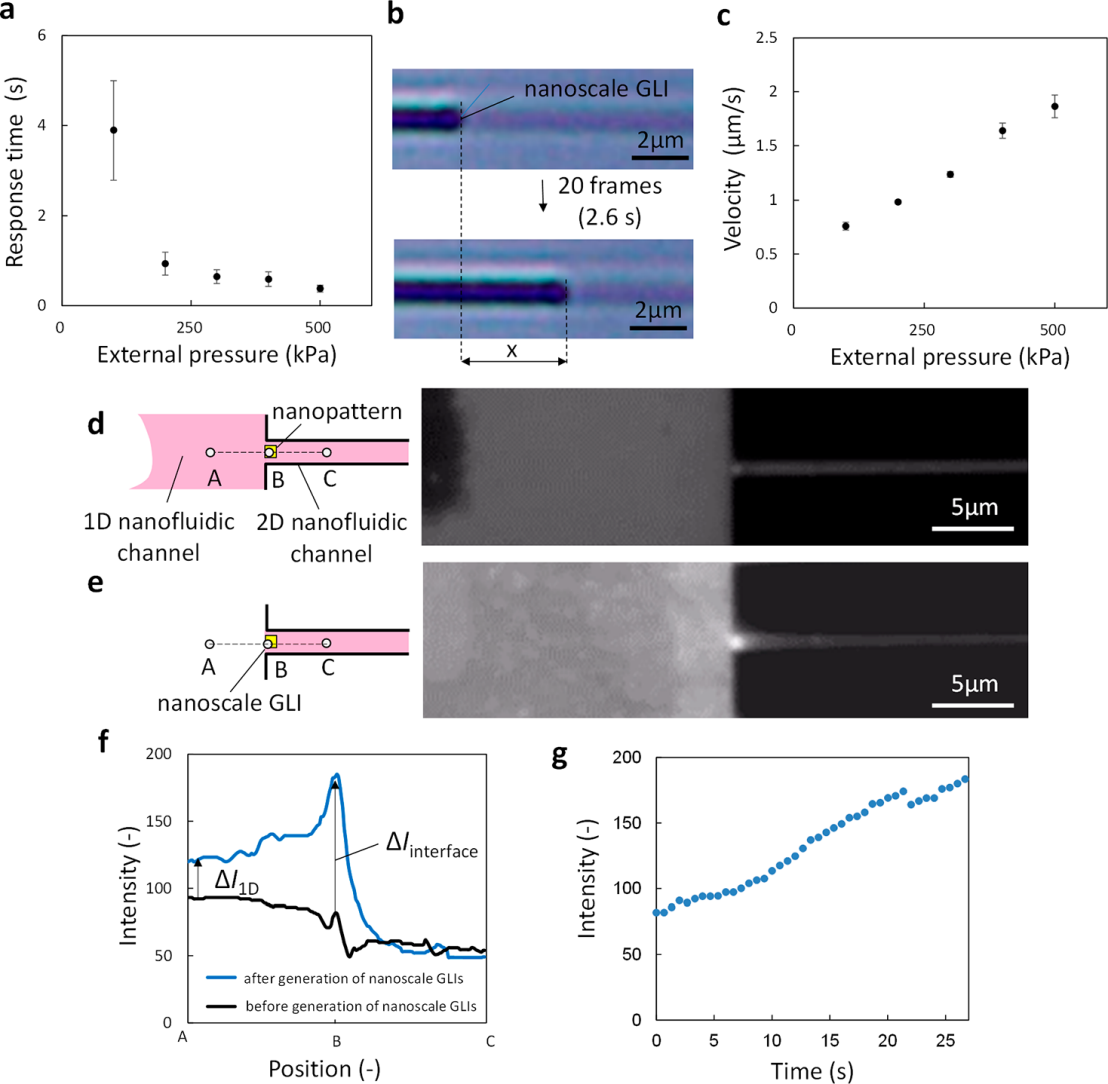
(a) Response time to move nanoscale GLIs at different external pressures. (b) Microscopic images of moving nanoscale GLIs in 2D nanofluidic channels. (c) Velocity of moving nanoscale GLIs at different external pressures. Schematic image and fluorescence image of (d) the rhodamine B solution-filled nanofluidic channels and (e) the fabricated nanoscale GLI in the 2D nanofluidic channel. (f) Fluorescence intensity profile, that is, fluorescence intensity between positions A and C in (d,e). (g) Change in fluorescence intensity at the position B in the 2D nanofluidic channel.

Enrichment is an important process for a wide range of chemical and biological applications. In the field of chemistry, GLIs have been widely applied as an effective and efficient method to enrich solute molecules from solutions. To demonstrate enrichment using nanoscale GLIs, an aqueous solution of fluorescent molecules (rhodamine B) was introduced in the nanofluidic device, and nanoscale GLIs of the solution were fabricated in the nanofluidic channels. The ability of nanoscale GLIs to enrich rhodamine B was evaluated by monitoring the change in the fluorescence intensity using an upright fluorescent microscope (Olympus BX53) with ultralong working distance objective lenses of high magnification (LUCPLFLN; Olympus) and a digital color camera (DP73; Olympus). The nanofluidic channels were filled with a solution of rhodamine B (1 M). After introduction of the solution into nanofluidic channels, the fabrication of nanoscale GLIs was initiated by applying an external pressure of 100 kPa (Figure 4d). After fabrication of nanoscale GLIs, the fluorescent molecules were enriched at the nanoscale GLIs (Figure 4e). In Figure 4d,e, the positions of A, B, and C were located at the 1D nanofluidic channels, nanopattern, and 2D nanofluidic channels, respectively. Figure 4f shows the fluorescence intensity profiles between A and C (Figure 4d,e). No significant difference in the black line between A and C indicates that the concentration of the solution was homogeneous in the nanofluidic channels before enrichment. The intensity of the blue line at B shows the solution of rhodamine B at the nanoscale GLIs. The difference of intensity around B (*ΔI*_interface_) suggests that nanoscale GLIs could enrich solute molecules. No difference of intensity between black and blue lines around C indicates that the concentration of rhodamine B was constant in 2D nanofluidic channels far from the nanoscale GLIs. This further supports the result of the enrichment at nanoscale GLIs. The blue line between A and B shows rhodamine B molecules in the dry 1D nanofluidic channels and has a larger intensity than the black line. The difference of intensity around A (Δ*I*_1D_) suggests that the molecules were adsorbed on the surface of the 1D nanofluidic channel. *ΔI*_interface_ is larger than Δ*I*_1D_, indicating that the enrichment at nanoscale GLIs dominated the process over the nonspecific adsorption. Figure 4g shows the change in fluorescent intensity at position B before (Figure 4d) and after (Figure 4e) the fabrication of nanoscale GLIs. The intensity linearly increased with time and doubled within tens of seconds. These results demonstrate that the enrichment was very quick at nanoscale GLIs.

In this paper, we report the fabrication, characterization, and application of the nanoscale GLIs by using nanofluidics. The nanoscale GLIs were fabricated through an approach involving a combination of precise local surface modification of nanofluidic channels, tailored physicochemical effects in nanospace, and optimized nanofluidic operations. The evaluation indicated that the fabricated nanoscale GLIs were uniform, arrayable, and stable. In addition, the nanoscale GLIs were manipulatable by applying external pressures and could be applied to the enrichment of molecules of interest at the nanoscales. We believe that the features and abilities of the fabricated nanoscale GLIs are quite favorable for not only the elucidation of mechanism of the nanoscale GLIs but also in the development of chemical, physical, and biological processes at the nanoscales in the future.

## Supporting Information Available

Methods and Figures S1–S7 showing the reflected light images of nanofluidic channels after bonding, the schematic drawing of the experimental setup for liquid introduction into nanofluidic device, the reflected light images of nanofluidic channels before, during, and after the surface modification process, the transmitted light images of water in surface-modified 2D nanofluidic channels under external pressure, results of water contact angle measurements on glass and gold surface before and after surface modification, and the schematic drawing of the nanofluidic control process for fabrication of nanoscale GLIs and the difference in luminance intensities between Figures 3h and 3i (PDF)
