# Supplementary material for: Fabrication of Nanoscale Gas–Liquid Interfaces in Hydrophilic/Hydrophobic Nanopatterned Nanofluidic Channels
Source: Nano Lett. 2021 Oct 14;21(24):10555–61. doi: 10.1021/acs.nanolett.1c02871 (PMC10860204; doi:10.1021/acs.nanolett.1c02871)
Supplement: Supplementary file 1 — nl1c02871_si_001.pdf [file nl1c02871_si_001.pdf]

Supporting Information

Fabrication of nanoscale gas-liquid interfaces in hydrophilic/hydrophobic nanopatterned nanofluidic channels

Hiroto Kawagishi,[1] *Shuichi Kawamata,*[2] *and Yan Xu*[1,3,4]*

[1]Department of Chemical Engineering, Graduate School of Engineering, Osaka Prefecture University, 1-2, Gakuen-cho, Naka-ku, Sakai, Osaka 599-8570, Japan

[2]Department of Quantum and Radiation Engineering, Graduate School of Engineering, Osaka Prefecture University, 1-2, Gakuen-cho, Naka-ku, Sakai, Osaka 599-8570, Japan

[3]Japan Science and Technology Agency (JST), PRESTO, 4-1-8 Honcho, Kawaguchi, Saitama 332-0012, Japan

[4]NanoSquare Research Institute, Research Center for the 21st Century, Organization for Research Promotion, Osaka Prefecture University, 1-2, Gakuen-cho, Naka-ku, Sakai, Osaka 599-8570, Japan

[*] Corresponding author: (Y. Xu) E-mail: xu@chemeng.osakafu-u.ac.jp

METHODS

Device fabrication

The nanofluidic device was fabricated based on previously reported protocols.[1–3] Nanofluidic channels were fabricated on a glass substrate (Sendai Quartz, Japan) using an electron beam lithography exposure system (ELS-7500EXI, ELIONIX, Japan), followed by dry etching with a plasma etching system (RIE-10NR, SUMCO, Japan) and vapor deposition using a vapor deposition machine (Seinan Industries, Japan). Microfluidic channels for liquid introduction were fabricated on a glass substrate using a milling machine (KitMill RD300, ORIGINALMIND, Japan). Subsequently, the two substrates were bonded, using previously reported low-temperature bonding techniques.[3,4]

Surface modification of open substrates

Nanochannels with identical surfaces were fabricated by baking four clean glass substrates (Sendai Quartz, Japan) as per reported fabrication protocols for similar nanofluidic devices.[1–3] Two substrates were coated with Cr (99.7 %, Nilaco, Japan) (5 nm) and Au (99.9 %, TANAKA Precious Metals, Japan) (45 nm) by vapor deposition. Further, we silanized the coated and uncoated substrates, by immersing these in a mixture of trichloromethylsilane (98 %, FUJIFILM Wako Pure Chemical, Japan) (47.5 μL), hydrochloric acid (36 %, Guaranteed Reagent, FUJIFILM Wako Pure Chemical, Japan) (125 μL), and toluene (99.5 %, Guaranteed Reagent, FUJIFILM Wako Pure Chemical, Japan) (10.0 mL) for 2 h, according to the conventional surface modification process.[5] Subsequently, one uncoated substrate and one gold-coated substrate were sequentially immersed into toluene, ethanol (99.5 %, Guaranteed Reagent, Nacalai Tesque, Japan), a 1:1 mixture of

ethanol and ultrapure water (Smart 2 pure, Thermo Fisher Scientific, USA), and ultrapure water for several seconds. The substrates were dried with nitrogen gas, followed by heating on a hot plate (HHP-441V, AS ONE, Japan) for 5 min at 100 °C. The remaining two silanized substrates were immersed in toluene for 24 h, followed by the above mentioned rinsing and drying treatments. We measured the contact angles of the four substrates using a contact angle meter (DM500, KYOWA, Japan) (Figure S5).

Surface modification for nanofluidic channels

Liquids filtered through 200 nm filters (Minisart, Sartorius Stedim Biotech, France) were pumped through the microfluidic channels and introduced into the nanofluidic device, by applying external pressure using a pressure controller (OBIMK3+, Everflow, France) (Figure S2). A mixture of trichloromethylsilane, hydrochloric acid, and toluene was passed into the nanofluidic device with an external pressure of 100 kPa for 2 h (Figure S3 b). We removed the molecules adsorbed on the Au surface, by rinsing the nanofluidic channels with toluene under an external pressure of 500 kPa for 24 h, followed by sequential rinsing with toluene, a 1:1 mixture of ethanol and ultrapure water, and ultrapure water for 1 h with an external pressure of 200 kPa. Lastly, the device was dried by heating for 5 min at 100 °C (Figure S3 c).

Measurement of Laplace pressure in modified nanofluidic channels

We introduced ultrapure water into the nanofluidic device at an external pressure of 100–500 kPa. Movement of gas-liquid interfaces (GLIs) in the 2D nanofluidic channels was observed using an optical microscope (BX53, Olympus, Japan) equipped with a CCD camera (DP73, Olympus,

Japan). The applied external pressure that moved the water in the 2D nanofluidic channels was treated as the Laplace pressure (Figures S4 a, b).

Fabrication of nanoscale GLIs

A 1:1 mixture of ethanol and ultrapure water was introduced into the hydrophobic nanofluidic device from the left microfluidic channels, under an external pressure of 500 kPa (Figure S6 b). We closed the entry of microfluidic channels and filled the mixture via the right microfluidic channels in the nanofluidic channels (Figure S6 c). We removed the liquids by purging air via the left microfluidic channels, at an external pressure of 200 kPa (Figure S6 d). Subsequently, the external pressure was raised from 0 to 500 kPa in increments of 100 kPa, to form nanoscale GLIs in the nanofluidic channels (Figure S6 e). The enrichment with nanoscale GLIs was performed using a 1 M solution of rhodamine B (Guaranteed Reagent, Nacalai Tesque, Japan) in 1:1 mixture of ethanol and ultrapure water, for generating nanoscale GLIs.

Data acquisition and analysis

Scanning electron microscopy of nanochannels and gold nanopatterns was performed using a field emission scanning electron microscope (SU8010, Hitachi High-Tech, Japan). We captured the events occurring in the nanofluidic device through an optical microscope equipped with a CCD camera. Images were analyzed using ImageJ software (NIH, Bethesda, MD, USA). Particularly, the image in Figure 4 e was processed with ImageJ to remove effects of photobleaching.

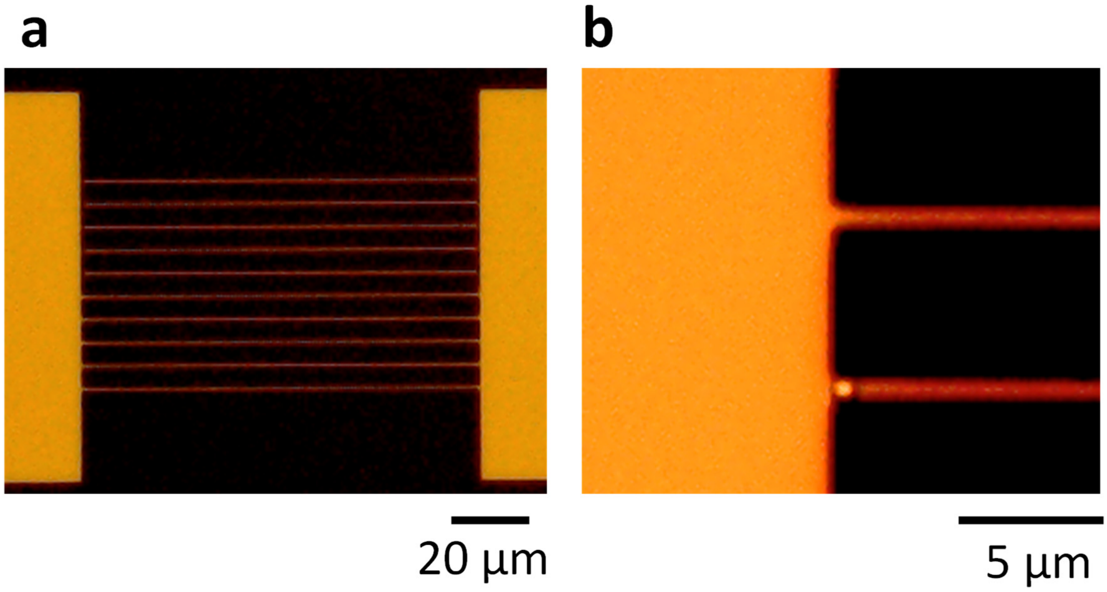

Figure S1. Microscopic images of (a) 2D nanofluidic channels between 1D nanofluidic channels after bonding; and (b) 2D nanofluidic channels with and without gold nanopattern after bonding.

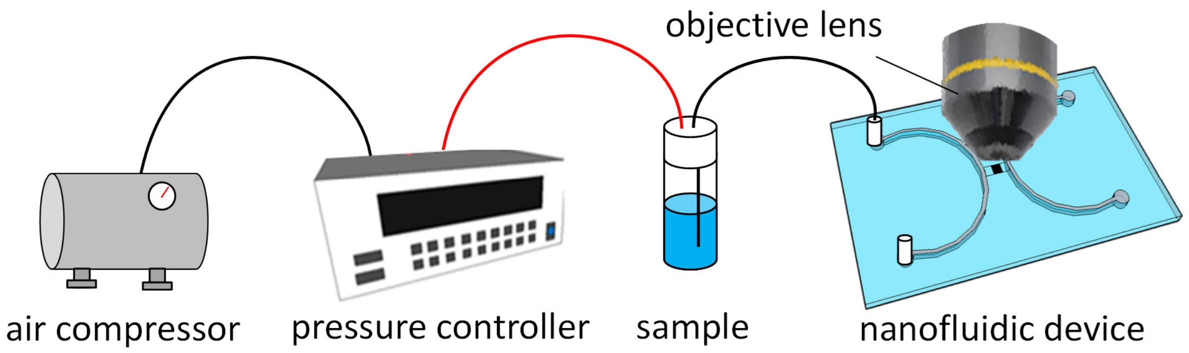

Figure S2. Schematic drawing of experimental setup including a nanofluidic control system and a microscope.

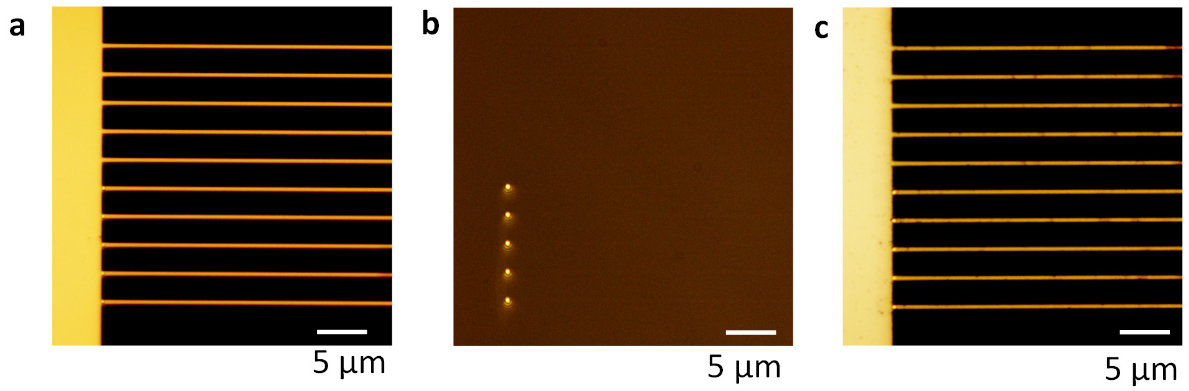

Figure S3. Microscopic images of nanofluidic channels (a) before, (b) during, and (c) after surface modification.

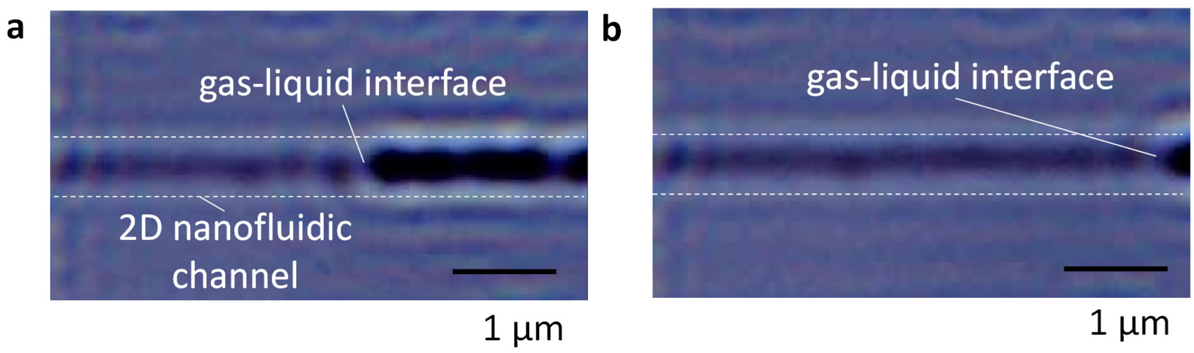

Figure S4. Microscopic images showing the introduction of water into 2D nanofluidic channels after surface modification, under an external pressure of (a) 400 kPa and (b) 500 kPa.

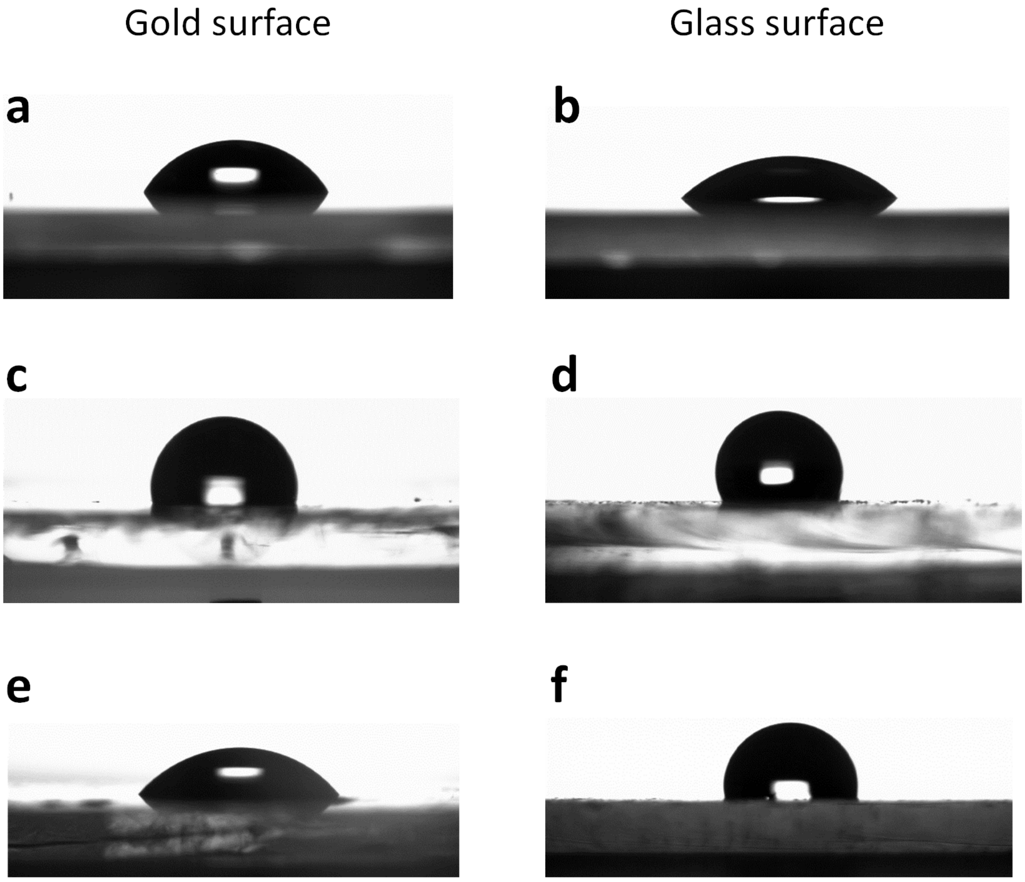

Figure S5. Water contact angle measurements on the gold coated and uncoated glass substrates (a, b) before surface modification; (c, d) after surface modification; and (e, f) surface modification followed by rinsing with toluene for 24 h.

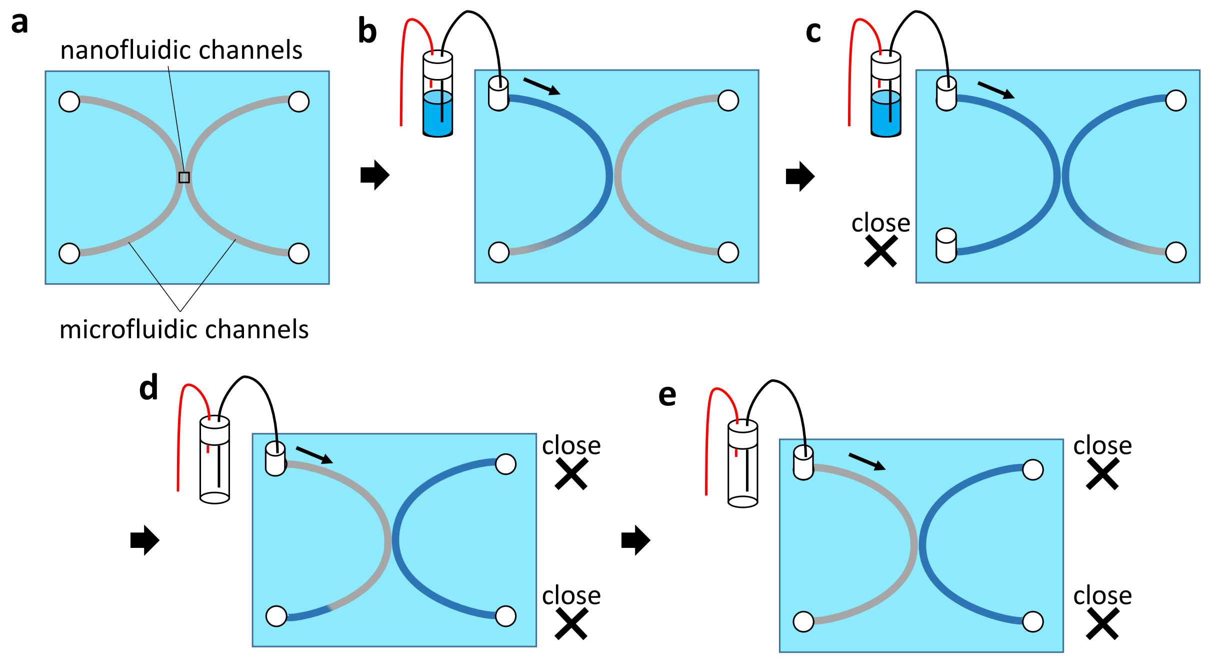

Figure S6. Nanofluidic control process for the fabrication of nanoscale GLIs in nanofluidic channels. Schematic drawing of (a) dry nanofluidic device; (b) filling liquid into the left microfluidic channels and nanofluidic channels; (c) filling liquid into the right microfluidic channels; (d) filling air into the left microfluidic channels for removing the liquid from microfluidic channels; (e) introducing air into the left microfluidic channels for the fabrication of nanoscale GLIs in nanofluidic channels.

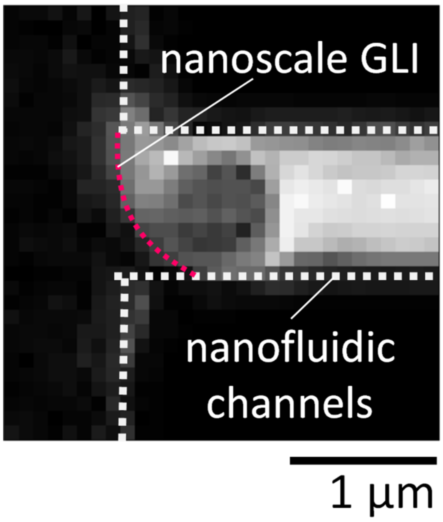

Figure S7. Difference in luminance intensities between Figures 3h and 3i, revealed by image processing using ImageJ.

REFERENCES

(1) Xu, Y.; Shinomiya, M.; Harada, A. Soft Matter-Regulated Active Nanovalves Locally Self-Assembled in Femtoliter Nanofluidic Channels. *Adv. Mater.* **2016**, *28* (11), 2209–2216.

(2) Xu, Y.; Wu, Q.; Shimatani, Y.; Yamaguchi, K. Regeneration of glass nanofluidic chips through a multiple-step sequential thermochemical decomposition process at high temperatures. *Lab Chip* **2015**, *15* (19), 3856–3861.

(3) Xu, Y.; Matsumoto, N.; Wu, Q.; Shimatani, Y.; Kawata, H. Site-specific nanopatterning of functional metallic and molecular arbitrary features in nanofluidic channels. *Lab Chip* **2015**, *15* (9), 1989–1993.

(4) Xu, Y.; Wang, C.; Li, L.; Matsumoto, N.; Jang, K.; Dong, Y.; Mawatari, K.; Suga, T.; Kitamori, T. Bonding of glass nanofluidic chips at room temperature by a one-step surface activation using an O2/CF4 plasma treatment. *Lab Chip* **2013**, *13*, 1048–1052.

(5) Wong, J. X. H.; Yu, H. Z. Preparation of transparent superhydrophobic glass slides: Demonstration of surface chemistry characteristics. *J. Chem. Educ.* **2013**, *90* (9), 1203–1206.
